# COVID-19 and breastfeeding: can SARS-CoV-2 be spread through lactation?

**DOI:** 10.15190/d.2021.11

**Published:** 2021-06-30

**Authors:** Radu Marian Florea, Camelia Madalina Sultana

**Affiliations:** ^1^Carol Davila” University of Medicine and Pharmacy, Bucharest, Romania; ^2^Stefan S. Nicolau Virology Institute, Bucharest, Romania

**Keywords:** Breastfeeding, COVID-19, newborn, pregnancy, SARS-CoV-2

## Abstract

SARS-CoV-2 is a new betacoronavirus that was first reported in the Hubei province, China, in December 2019. The virus is likely transmitted through air droplets. However, there are reported cases where SARS-CoV-2-RNA was found in other samples, such as blood or stool. Nonetheless, there is limited information concerning the presence of viral RNA in pregnancy-related samples, specifically breast milk. However unlikely, there is still uncertainty regarding the possibility of vertical transmission from mother to infant through breastfeeding. This review aims to synthetize the literature written so far on this topic. Despite not being extensively researched, vertical transmission through breast milk seems unlikely. Case series showed that milk samples from mothers with COVID-19 were almost entirely negative. So far, there have been only 9 recorded cases of viral shedding in milk samples, uncertain however of the viability of the particles. Furthermore, WHO and UNICEF strongly encourage commencing breastfeeding after parturition, underlining the benefits of lactation. Moreover, some studies have proven the existence of IgG and IgA anti-SARS-CoV-2-antibodies in the maternal milk that could possibly play an important part in the neonate’s protection against the virus. Vertical transmission through lactation seems unlikely, most studies pointing towards the safety of breastfeeding. However, further larger-scale studies need to be performed in order to clarify a yet uncertain matter.

## SUMMARY

1. *Introduction*
2. *What could we learn from the past?*
3. *Vertical transmission in SARS-CoV-2; What we know so far?*
4. *Benefits and disadvantages of breastfeeding*
5. *Approach towards breastfeeding during COVID-19 pandemic*
6. *Conclusion*

## 1. Introduction

Severe acute respiratory syndrome-coronavirus 2 (SARS-CoV-2) is a new betacoronavirus, a positive-sense single-stranded RNA virus, member of the Coronaviridae family, closely related to other severe coronaviruses, such as the Severe acute respiratory syndrome coronavirus/SARS-CoV (sharing 76.47% similarities^1^), which was responsible for an outbreak in 2003, and Middle East respiratory syndrome/MERS-CoV (50% similarities^2^), that produced an outbreak in 2012. However, the amplitude of the SARS-CoV-2 outbreak largely exceeded its predecessors, so much so that on March 11, 2020, the World Health Organization declared it a pandemic. The first cases were reported in Hubei province, China with the epicenter around a raw sea-food market in Wuhan^3^. Ever since, the virus has spread uncontrollably worldwide, affecting almost 173 million people, among whom almost 3.7 million have died^4^. The disease produced by SARS-CoV-2 is known as Corona Virus Disease 19 (COVID-19), and is presented in numerous manners, most common symptoms being^2,5^. fever (85.6%), headache, cough (65.7%), muscle ache, tiredness (42.4%). Other disorders reported are: diarrhea, loss of smell/taste, dyspnea (33%) and pneumonia, acute respiratory distress syndrome or myocarditis in more severe cases^6-8^. Usually, in healthy young people COVID-19 has a mild symptomatology, whereas, in elderly, the case fatality can get up to 14.8% in the case of people of 80+ years old^9^. The receptor that SARS-CoV-2 uses in the process of internalization is the ACE2 receptor^10^, similarly to SARS-CoV. This receptor is found on cells belonging to almost all organs, including heart, arteries, small intestine, hypothalamus etc^10^. However, type 2 pneumocytes from lungs have the highest concentration of ACE2 receptors^10^. So far, the main means of transmission of the virus is through droplets of saliva or nasal discharge^11^, thus favoring close contact and familial clusters spreading. In order to limit the spreading of the virus, the major public health organizations have urged people to wear surgical face masks when in enclosed spaces and to try and avoid crowded areas as much as possible, while practicing social distancing^12^. However, there have been reported cases where SARS-CoV-2 RNA has been found in other samples, such as: blood, urine, saliva, stool etc^13^. Nonetheless, there is limited information concerning viral RNA presence in pregnancy-related samples (i.e. amniotic fluid, placenta, umbilical cord blood, vaginal samples etc.), specifically in breast milk. However unlikely, there is still uncertainty regarding the possibility of vertical transmission from mother to infant via breastfeeding. The aim of this review is to synthetize the literature written on this topic, while presenting the data in a more compact way.

A systematic review of the literature was performed, mainly on online databases, such as PubMed, Scopus, Embase, targeting articles concerning pregnant women with COVID-19, infants born of COVID-19 positive mothers, as well as postpartum women infected with SARS-CoV-2.

## 2. What could we learn from the past?

Being a new virus, there is still much to learn about SARS-CoV-2 and the way it is spread. So far, there is relatively scarce research concerning the possibility of vertical transmission of the virus from mother to infants. However, in order to get a stronger picture regarding this issue we could look to the past and study the behavior of other viruses in connection with postpartum or perinatal transmission and compare the information with what we know so far about SARS-CoV-2. Several other viruses that can be taken into consideration are human immunodeficiency virus (HIV)^14^, that is known to be transmitted through breastfeeding, and the other two previous coronaviruses that we faced, SARS-CoV^15,16^ and MERS-CoV^17,18^.

The HIV was first isolated in 1983 by two different teams from France^19^ and the United States. Even though initially it was associated exclusively with LGBTQ+ community or drug addicts, it was later proven that everybody is susceptible to this virus. In 1985 the first case of vertical transmission of HIV from mother to infant was reported^20^, being suspected that the means of transmission was via breastfeeding, later that year the first samples of maternal milk in which the HIV RNA was found were reported, advancing the theory that the disease can be spread through breast milk. Afterwards, most international health organizations advised mothers who were infected with HIV to avoid breastfeeding and instead opt for formula-based milk^21^. However, in the long run it was proven that it was a difficult issue to tackle, since not all countries and not all people had the possibility to afford formula-based diets for the infants. Thus, developing countries, especially from the African continent, were faced with a social and epidemiologic conundrum: shifting the diet towards formula-based feeding would help in preventing the spread of HIV, but would affect the development of the babies and increase the rate of infantile mortality, while keeping breastfeeding as the standard choice would help in the development of the babies, but would also increase the spreading rate of HIV^22^. Nowadays, in developed countries it is still recommended to pursuit formula feeding in the case of women infected with HIV. However, in developing countries the paradigm has shifted once again, cautiously encouraging breastfeeding^23^.

Middle East respiratory syndrome coronavirus (MERS-CoV) is a betacoronavirus related to SARS-CoV-2 that produced an outbreak in 2012. The first cases have been reported in Saudi Arabia in 2012 and are supposed to have been transmitted from infected camels^24^. Person-to-person transmission is facilitated by close contact. So far MERS is the deadliest of the coronavirus-related diseases with a mortality rate of almost 36%. The common symptoms of MERS are fever, cough, chills, myalgia and shortness of breath in mild cases, while in severe cases patients develop acute respiratory distress syndrome^25^. However, it is considered that the fatality rate is slightly lower, due to possibly missing asymptomatic or paucisymptomatic cases, especially in developing African countries. There have been no reports concerning the presence or absence of MERS-CoV in human milk. Albeit not many, the few pregnant women infected with MERS gave birth to healthy children, negative at the RT-PCR tests for the virus^26,27^. However, there are reported cases where samples of camel milk have been tested positive for MERS-CoV nucleic acid^17,18,28^ and even one case when a person got infected with MERS after consuming raw camel milk^29^. Therefore, the authorities have urged people to cautiously consume camel milk and to always respect the sanitary regulations, such as boiling/pasteurizing the milk.

Severe acute respiratory syndrome coronavirus (SARS-CoV) is also a betacoronavirus, the most related to SARS-CoV-2 (76.47% similarities^1^). SARS-CoV was first discovered in 2002 in the province of Guandong China, being traced back as well to a “wet” market^30^. The outbreak has lasted until the summer of 2003, being successfully contained in only 7-8 months^31^. Overall it has affected more than 8400 people, killing 916 of them, thus having a mortality rate of almost 10%^31^. The most common symptoms of the disease are: fever, chills, cough and myalgia. Lymphocytopenia, mild thrombocytopenia and an increase in the level of D-dimers are common laboratory findings in SARS^32^. The main way through which SARS-CoV spreads is through air droplets, thus favoring person-to-person and familial clusters spreading. So far, there is no study demonstrating SARS vertical transmission from mother to infant. There has been only one report to have assessed milk samples, failing to find any trace of SARS-CoV RNA^15^. However, in that same article by Robertson et al. in the milk samples antibodies that are believed to have a potential immunogenic effect were found^15^. Therefore, given the scarce evidence on this topic it is difficult to properly assess whether we could talk about vertical transmission or viral shedding in milk samples in the case of the SARS-CoV.

## 3. Vertical transmission in SARS-CoV-2. What we know so far?

There is still very much to learn about SARS-CoV-2 and its mechanisms. Of particular interest to this article is assessing the data that we have so far concerning the potential of vertical transmission of the virus from mother to infant via breast milk. There have been several articles written on this topic and at the time being, the general consensus seems to lean towards not considering the spread of the virus via lactation a likely scenario. However, there have been reported 12 cases where SARS-CoV-2 RNA was found in milk samples^33-44^. It is still uncertain, however, whether the viral particles were capable of replication and thus, of infecting the infants (see Table 1).

**Table 1:** Reported SARS-CoV-2 positive milk sample cases

Publication	Number of positive breast milk samples	Other maternal positive samples	Infant SARS-CoV-2 RT-PCR result	Other neonatal positive samples	In favor/against breastfeeding
Wu et al.^33^	1/3	No	Negative	No	Rather against
Zhu et al.^34^	3/3	No	NA	NA	Rather against
Bastug et al.^41^	3/3	No	8 hours- negative 96 hours-positive	Stool-positive (+96 hours); Serum-positive (+96 hours)	Cautiously against
Groß et al.^38^	4/7	No	Yes, 3 days after mother	Respiratory sincitial virus-positive	Neutral, demand larger studies
Fenizia et al.^43^	1/32	IgM antibodies in milk	Negative	N/A	Neutral
Costa et al.^39^	3/6	Cord blood, placenta	NA	NA	Cautiously-against, demand testing samples before feeding infants
Tam et al.^37^	2/7	No	Yes	Stool-positive	Cautiously in favor
Lugli et al.^36^	2/2	No	Negative	No	Cautiously in favor
Chambers et al.^40^	1/66	NA	NA	NA	In favor
Bertino et al.^35^	1/14	Breast milk tested positive again 26 days after parturition	Yes, at 2 days Negative on days 16, 26, 38	No	In favor
Hinojosa-Velasco et al.^44^	1/2	Stool samples	Positive immediately after birth	Stool samples	In favor. Raise concerns regarding in-utero vertical transmission.
Thanigainathan et al.^45^	1/31	No	Negative	No	In favor

As would have been expected, the first situations of reported viral shedding in milk samples came from China, the origin point of the pandemic. In a case series based on 5 pregnant women infected with SARS-CoV-2, Zhu et al.^34^ have discovered two samples of breast milk with viral shedding from the same patient. Even though they underline the importance of feeding infants with fresh milk, given its benefic effects on the neonate’s development, they adopt a rather cautious stance regarding breastfeeding in the case of women infected with SARS-CoV-2, pointing out that it is still unclear how usual this situation could be or whether the viral particles were viable or not. However, the authors recommend further research on larger cohorts in order to get a broader perspective on this topic. A similar stance is taken by Wu et al.^33^ who have also reported one breast milk sample to be positive for SARS-CoV-2 RNA. However, after a couple of days, the milk samples of the same woman turned negative. Moreover, all the babies were repeatedly tested negative for SARS-CoV-2.

Another case was reported in Turkey^41^, where samples of milk from 8, 72 and 96 hours after parturition were tested positive for SARS-CoV-2 RNA. An initial test for the infant performed around 8-10 hours after birth retrieved a negative result. However, after 96 hours, another nasopharyngeal swab has been sampled and the result this time was positive, so were the stool samples after 96 hours. The authors therefore advance either the possibility of viral transmission through the milk consumed by the infant during the first hours, or the possibility of getting infected during parturition with an initial false negative result after 8 hours from birth, advising parents to carefully discuss the issue with their healthcare providers in order to agree whether the benefits of breastfeeding truly outweigh the potential risks. Likewise, another case of milk samples that tested positive for SARS-CoV-2 RNA was reported in Italy by Costa et al^39^. During her stay in the hospital, the woman got 3 positive milk samples out of a total of 6 and also specimens from her umbilical cord blood and placenta retrieved positive result at the RT-PCR test. However, the authors have concluded that, in the future, breast milk samples should be tested before commencing lactation. It also pointed out the need for larger studies that could clarify whether or not vertical transmission via maternal milk is a common means of spreading the virus. Furthermore, a case from Germany reported by Groß et al.^38^ found positive milk samples on 4 consecutive days. After breastfeeding, the infant was tested positive for COVID-19 as well. The authors suggest that it is still unclear whether the baby got infected because of the maternal milk or because of being in close contact with the SARS-CoV-2-positive mother.

Tam et al.^37^ have also documented a case of a mother and her 2-month old infant that have returned to Australia from an endemic COVID-19 area. The mother was first admitted to hospital for mild respiratory symptoms, followed by the baby, just one day later. During the stay in hospital, two samples of breast milk were tested positive for SARS-CoV-2 RNA. However, the authors couldn’t precisely state whether the particles found were viable or not. Given the positive evolution of the two, even after resuming lactation, Tam et al. have concluded that COVID-19 transmission through milk is rather unlikely and the advantages of breastfeeding probably outweigh the risks. Nonetheless, the authors underline the need for further assessment regarding the viability of RNA particles found in maternal milk.

Hinojosa-Velasco et al.^44^ have also published a case report describing the situation of a woman from Mexico whose newborn daughter tested positive for SARS-CoV-2 RNA from a nasopharyngeal swab immediately after parturition. Therefore, the authors raise concerns regarding the possibility of an in-utero vertical transmission. In what it concerns the milk samples, they consider the likelihood of disease spreading through this means rather improbable, concluding that the potential benefits of breastfeeding, such as the presence of IgM and IgA antibodies, greatly outweigh the risks. Similarly, Bertino et al.^35^ have concluded that the spread of the virus via breastfeeding is rather unlikely, although viral shedding in milk samples could be possible, albeit unlikely. The authors have documented the cases of 14 pregnant women infected with SARS-CoV-2 whose breast milk samples have been tested, only one sample yielding a positive result. The infant of that woman eventually got tested positive as well, but the authors consider that the transmission was either intra-partum or horizontal after birth.

In Italy, a case series conducted by Fenizia et al.^43^ analyzed the samples collected from 32 women infected with SARS-CoV-2. The authors have reported 2 cases of possible congenital infection with maternal placental and cord blood samples tested positive for SARS-CoV-2 RNA, as well as the neonates’ nasopharyngeal swabs yielding positive results. These are the only two neonatal positive tests recorded. Moreover, a milk sample from one of the patients was also tested positive for SARS-CoV-2 RNA. However, the woman whose breast milk contained viral RNA had no other sample tested positive and her infant’s nasopharyngeal swab also provided a negative result. In the breast milk of the same woman were also found IgM antibodies.

In a case series published by Thanigainathan et al.^45^, milk samples from 30 SARS-CoV-2 positive women, as well as nasopharyngeal swabs from their infants were tested for viral RNA presence. Out of all the milk samples, only one yielded a positive result. However, another breast milk sample collected from the same woman retrieved a negative result the following day. No infant nasopharyngeal swab was tested positive, and all mothers were encouraged to room-in with their babies, as well as breastfeed them, while strictly abiding to some safety regulations. Due to the fact that no infant got infected, despite the one milk sample that was tested positive, the authors concluded that viral spreading via breast milk is rather unlikely, and therefore, mothers should be encouraged to breastfeed and maintain close contact with the babies.

Nonetheless, one of the most enlightening studies up to date belongs to Chambers et al.^40^, who have conducted a research study between March 27 and May 6, 2020, in which they evaluated 64 milk samples from 18 women who have been tested positive for SARS-CoV-2. Out of all these samples, only one tested positive for the virus. However, even that sample was not replication competent. Therefore, the authors suggest that, given the small number of cases where the milk samples are tested positive for SARS-CoV-2, corroborated with the incapacity of replication of those samples, vertical transmission of the disease through maternal milk is rather unlikely and therefore the process of breastfeeding should be encouraged, provided the safety measures are respected^46^.

In the June of 2020, WHO points out that, although there have been reported a few cases where SARS-CoV-2 RNA was discovered in breast milk samples, it is unclear whether the viral particles were replicative or infective. Therefore, WHO recommends further studies concerning the replication capacity of the viral particles found in milk, as well as more tests assessing infectivity in animal models before drawing definitive conclusions. Unless additional studies that contradict the existing consensus are published, WHO currently strongly encourages asymptomatic and paucisymptomatic women to breastfeed and to maintain close contact with their infants, while strictly abiding to several safety measures^46^.

The vast majority of reports concluded that, given what is known so far, there is a small likelihood that COVID-19 could be spread through breastfeeding. Studies conducted so far discovered that the rate of vertical transmission from mother to infant is of only 4.2%, therefore rather unlikely^47^. Overall, 29 articles documenting the cases of 98 women, assessing the presence of SARS-CoV-2 RNA in milk samples using RT-PCR testing, failed to evidence viral shedding in the specimens. The cases are presented synthetically in Table 2. In regard to geographical distribution, out of the total number of researches, there are: China (20 articles)^48-67^, Italy (3 articles)^68-70^, Republic of Korea^71^, Spain^72^, Belgium^73^, Singapore^74^, Jordan^75^ and Turkey^76^. Out of all these cases, there have been reported 10 infants that were tested positive for SARS-CoV-2 (10/98), 28 infants that were not breastfed (28/98), 18 neonates that were breastfed (18/98), while there is nothing mentioned about the remaining cases. The general consensus therefore, seems to underline the unlikelihood of vertical transmission via breast milk^77^. However, it is universally agreed among the researchers that further studies need to be conducted.

A particularly odd situation was described by Dong et al.^50^in a case report concerning a pregnant woman infected with SARS-CoV-2. After delivery, the neonate was tested for COVID-19 and the results were negative on 4 different occasions. Moreover, the milk and vaginal samples were also negative. However, right after parturition, in the serum of the neonate, IgM antibodies were evidenced. It is known that IgM antibodies can’t cross the placental barrier and are only produced by the fetus in utero, therefore pleading for a congenital infection with SARS-CoV-2.

Yan et al.^57^ have conducted a case series concerning 116 neonates overall. Among them, 86 infants have been tested for COVID-19 using RT-PCR technology, all of the samples being negative. Despite only testing 12 breast milk samples, all of them yielding negative results, it is safe to assume that vertical transmission via breast milk is rather unlikely, since none of the neonates was diagnosed with COVID-19.

In an article written by Pace et al.^78^, milk samples and breast swabs from 18 women have been assessed for viral RNA presence. Out of the 37 milk specimens, none was tested positive. Furthermore, all the samples contained IgG and IgA antibodies that have been proven to possess neutralizing effects against the virus (62% of the samples accomplished neutralizing effect). During this research, 70 breast swabs have been collected as well, both before and after washing the area. One specimen sampled before sanitizing the breast has yielded positive results. However, the specimen collected from the same person after washing the breast retrieved negative results, thus proving that, provided the hygiene measures are respected, breastfeeding should not lead to vertical transmission of the virus.

A recent longitudinal study^58^ conducted in Hubei assessed the effect that the pandemic had on the process of breastfeeding among mothers who were infected or suspected of COVID-19. Throughout the research, 44 samples of breast milk were analyzed and none of them proved to be positive for SARS-CoV-2 RNA, thus signaling once again the unlikelihood of vertical transmission through lactation.

Another research that hasn’t explicitly evaluated milk samples with RT-PCR tests, but rather have infirmed the transmission of SARS-CoV-2 based on the absence of positive nasopharyngeal swabs taken from the infants breastfed by infected mothers was conducted by Pereira et al.^79^ where they assessed the evolution of 23 pregnant women and their neonates.

**Table 2:** Reported negative cases of milk samples

Publication	Number of subjects	Retested milk samples	Infected infant	Infant breastfed	Observations
Han et al.^71^	1	N/A	Yes	Yes	High viral load in infant’s tests. Viral presence in all samples (blood, urine, stool). Viral persistence in stool after symptoms cessation.
Kalafat et al.^76^	1	N/A	No	N/S	Pregnant woman with severe respiratory symptoms admitted to the ICU and intubated. Negative placental, cord blood and milk samples. Neonatal swab was negative.
Mao et al.^48^	1	Yes	Yes	Yes	Viral persistence in stool and nasopharyngeal swab after symptoms cessation. Presumed household horizontal transmission.
Peng et al.^49^	1	Yes	No	No	Besides maternal nasopharyngeal swab all other maternal and neonatal samples were negative. Breast milk samples negative on 7 different tests.
Xiong et al.^60^	1	No	No	N/S	Negative samples from milk, cervical secretion, amniotic fluid, rectal swab.
Chen et al.^61^	3	N/S	No	N/S	Milk samples from 3 women infected with SARS-CoV-2 yielded negative results.
Piersiglli et al.^71^	1	N/S	Yes	No, expressed milk used instead	Horizontally acquired COVID-19 in a preterm neonate with unspecific symptomatology.
De Socio et al.^68^	1	No	No	Yes	Apart from maternal nasopharyngeal swab all sample were negative.
Perrone et al.^69^	1	Yes	No	Yes	Milk samples negative on 3 occasions.
Zaghal et al.^75^	1	No	No	Yes	Neonatal nasopharyngeal swab was negative on 3 occasions.
Dong et al.^62^	1	Yes	Yes	Yes	IgG and IgA antibodies discovered in the breast milk.
Deng et al.^63^	6	N/S	No	N/S	Negative neonatal samples.
Lang et al.^64^	1	No	No	N/S	Apart from maternal nasopharyngeal swab, all other maternal and neonatal samples were negative.
Wang et al.^65^	1	No	N/S	N/S	Consistently positive maternal stool sample. Other maternal samples (milk, nasopharyngeal swab) were negative.
Chen et al.^66^	6	No	No	N/S	Apart from maternal NPS all other maternal and neonatal samples were negative.
Cui et al.^67^	1	Yes	Yes	Yes	Severe symptomatology in a 55 days old infant. Infant positive tests persist in stool samples and anal swabs after symptoms cessation. Maternal positive tests in anal swabs.
Dong et al.^50^	1	No	No	No	Negative maternal milk and vaginal samples. Consistent negative neonatal nasopharyngeal swab on 4 different occasions. IgM antibodies found in the neonate’s serum, thus potentially suggesting for in-utero vertical transmission.
Fan et al.^51^	2	Yes	No	No	Apart from maternal nasopharyngeal swabs, all other samples, both maternal and neonatal, were negative.
Kam et al.^74^	1	No	Yes	Yes	Persistently positive infant’s nasopharyngeal swab tests until day 16. First day-viremia corroborated with a fever episode. Initially stool samples were negative, later turned positive.
Li et al.^52^	1	Yes	No	N/S	Apart from maternal nasopharyngeal swabs, all other samples, both maternal and neonatal, were negative.
Salvatori et al.^70^	2	No	Yes	Yes	Neonatal nasopharyngeal swabs positive, presumed horizontal transmission; Breastfeeding allowed.
Liu et al.^53^	10	No	No	No	Apart from maternal nasopharyngeal swabs, all other samples, both maternal and neonatal, were negative.
Wang et al.^54^	1	No	Yes	No	Neonate tested positive 36 hours after birth. Cord blood, breast millk and placental samples yielded negative results. Vertical transmission can’t be ruled out.
Yu at al.^55^	1	Yes	Yes	Yes	Apart from nasopharyngeal swab, all other maternal samples were negative. Nasopharyngeal and stool samples consistently tested positive in the case of the infant. No SARS-CoV-2 RNA was found in the breast milk. However, IgG antibodies were detected, with a potential benefic impact for the infant’s development.
Lei at al.^56^	4	N/S	No	N/S	No other maternal positive sample apart from nasopharyngeal swabs. No neonatal samples were positive.
Yan et al.^57^	116 infants in total	No	No	N/S	86/86 neonates tested for COVID-19 had negative results. 12/12 milk samples were negative. 6/6 vaginal samples were negative. 10/10 amniotic fluid and cord blood samples were negative.
Marin et al.^72^	7	N/S	No	Yes	No infant was tested positive for SARS-CoV-2. All mother-infant dyads were allowed skin-to-skin contact.
Peng et al.^58^	25	Yes	No	Majority chose expressing milk	No milk sample tested positive. No vertical transmission via breast milk reported.
Gao et al.^59^	12	Yes	No	No	No viral shedding in maternal milk or cord blood. IgG and IgM detected in 3 breast milk samples.
Pace et al.^78^	37	No	N/S	N/S	37 milk samples tested negative for viral RNA. 1 positive breast swab turned negative after washing the skin; IgG and IgA antibodies found in milk samples.

Based on the World Health Organization recommendations^46^, mothers were allowed to breastfeed, as well as maintain skin-to-skin contact, as long as the safety measures were respected. No neonatal COVID-19 case was reported, therefore, the vertical transmission of the disease seems unlikely, provided the recommendations are respected.

In another study conducted in Spain, Marin et al^80^. have stated that no case of vertical transmission has been noticed. Out of the 42 neonates included in the review, only 3 of them were initially tested positive, only to get negative results just a day later, thus suggesting false positive results in the first place. Furthermore, 23.8% of the infants were breastfed from the beginning, the number climbing to 47.5% at discharge. None of the babies that were breastfed developed symptoms, nor got tested positive for SARS-CoV-2 infection, infirming therefore the vertical transmission theory.

Consistent with these discoveries, Lowe et al^81^. have documented a case report of a woman infected with SARS-CoV-2 that gave birth vaginally to a healthy infant. The neonate’s nasopharyngeal swab yielded negative results. Postpartum, the parents (mother and father), both tested positive for SARS-CoV-2 infection, insisted on being isolated in the same room with the infant and proceeded to breastfeed him. The precaution measures^46^ were respected at all times and the evolution of both mother and neonate was favorable, the family being discharged 4 days later. Therefore, the authors conclude that, as long as the safety precautions are respected, the scenario of vertical transmission from mother to infant via breast milk is rather unlikely.

So far, most research studies have failed to evidence the presence of SARS-CoV-2 RNA in breast milk, thus suggesting that vertical transmission via maternal milk is rather unlikely. Moreover, the benefits of breastfeeding have been documented long before the COVID-19 pandemic. Therefore, most authors, as well as the majority of health organizations, lean towards encouraging breastfeeding and skin-to-skin contact between mother and infant.

## 4. Benefits and disadvantages of breastfeeding

Currently, most studies acknowledge the benefits of breastfeeding and consider that the advantages vastly outweigh the potential risks, thus encouraging lactation for at least 6 months after parturition, both before and during the COVID-19 pandemic. Some of the benefits of breastfeeding concern both the well-being of the infant and the mother as well.

One of the most important aspects that support breastfeeding is the existence of anti-SARS-CoV-2 antibodies in the milk samples, having been evidenced by some research studies already. These antibodies could potentially play an important part in the development of the immune system of neonates and not only, some authors even taking into consideration the idea of using hyperimmune raw cow milk, for example, as a measure of short-term protection against the virus^82^.

Previously it has been proven that breast milk contains antibodies that protect the infant from many viruses, such as: syncytial respiratory virus^83^, influenza A virus^84^, rotavirus^85^ etc. Recently, several investigations have discovered anti-SARS-CoV-2 antibodies in maternal milk, thus emphasizing the potential benefic effect that breastfeeding could have in limiting the spread of the virus.

The first authors to report the presence of antibodies in milk samples collected from infected mothers were Yu et al.^55^, who have documented the case of a 13 months old infant tested positive for SARS-CoV-2 infection along with his mother, also positive for the virus. The parents have insisted on continuing breastfeeding, despite the maternal positive results. Milk samples have been collected and assessed for viral RNA, as well as antibodies presence. It was proven that the specimens were negative for viral shedding. However, there have been discovered IgG antibodies in 2 different samples. Therefore, the authors concluded that the presence of antibodies could potentially play an important part in the development of an immune response for the infant. Similar results have been reported by Dong et al^62^. who have discovered IgG and IgA antibodies in the breast milk of a woman infected with SARS-CoV-2. Furthermore, on 3 different occasions, the milk samples yielded negative results by RT-PCR test for viral RNA.

Consistent with these results, Gao et al^59^. have evaluated samples collected from 14 women infected with SARS-CoV-2. Out of all the samples collected, 3 breast milk specimens tested positive for IgG, while another tested positive for IgM antibodies. The authors point out that the antibodies discovered in the milk samples are similar to the ones used in convalescent plasma therapy. However, unlike plasma, breast milk is not allergenic and could therefore be used more easily to treat patients (especially infants) infected with SARS-CoV-2, the authors suggesting the need for further research in order to discover a way in which immune breast milk could be used as a means of treating infected patients. IgG antibodies presence in the milk samples was also reported by Preßler at al^86^. in a case series conducted during an outbreak in a German maternity. The samples collected from a woman infected with SARS-CoV-2 were tested positive for IgG antibodies.

In a research conducted by Fox et al^87^, presenting preliminary data from a much larger cohort, it has been proven that in 12/15 milk samples the IgA titer was much higher than expected. The author suggests that, if definitive results turn out to be consistent with the preliminary ones, breast milk could be used as a means of treating SARS-CoV-2 infected patients, especially infants, since secretory IgA is better suited for the respiratory mucosal environment. The study presents interest not only for the COVID-19 pandemic, but also for the future by advancing the knowledge and filling the existing gaps regarding breast milk immunology. Furthermore, the authors have noticed that IgA present in the milk also displays an unspecific cross-reaction to SARS-CoV-2 antigens, consistent with the discoveries of Demers-Mathieu et al.^88^, who have suggested that even milk samples from the mothers that were not previously infected with SARS-CoV-2 could provide a certain unspecific protection, due to cross-reaction with antibodies against other coronaviruses. Similarly, a case report conducted by Lebrao et al^89^. has emphasized IgA antibodies in the milk samples collected from an infected mother. The author suggests that these antibodies could have a benefic effect on the infant’s evolution, by contributing to the development of the immune system.

However, apart from the potential role of mitigating the severe symptoms of COVID-19 as well as limiting the spread of the virus, it has long been known that breastfeeding and skin-to-skin contact between mother and infant have much more benefits^90^. The experts suggest that exclusive breastfeeding should be applied for the first 6 months, followed by food and breastfeeding for the following year^91^.

Among the multiple benefits that maternal milk has on the infant’s development we can count protection from infectious diseases such as bacterial meningitis^92^, necrotizing enterocolitis^93^ etc. Moreover, breastfeeding has been associated, according to some authors, with reduced risk of developing leukemia^94^, asthma^95^, type 1 diabetes^96^, as well as a role in the development of the neonate’s cognitive abilities^96^ .

Moreover, breastfeeding has positive effects on maternal evolution as well, reducing the risk of developing breast or ovarian cancer postpartum^96^. Breastfeeding has also been associated with a decrease in the risk of postpartum depression among mothers^96^.

However, despite being considered the elective means of feeding an infant for at least the first 6 months of life, breastfeeding also presents some risks and contraindications. The most notable ones are a series of infectious diseases that could spread and infect the baby either by the milk per se, or by the close contact between the mother and the infant created in the process of lactation. Apart from some viral diseases, such as the HIV or HTLV infections, some other ailments have been deemed risky for breastfeeding, such as tuberculosis, varicella, brucellosis or herpes simplex virus lesions on the breast^91^. Moreover, in the case of women who breastfeed while being pregnant, there is a risk of affecting the composition of the postpartum milk, as well as the growth of the infant, especially in developing countries^97^. Furthermore, women who are using medications may be more prone to stopping breastfeeding early, due to potential adverse effects in the case of the baby. This aspect is accentuated by the fact that the use of 90% of medicine is restricted during pregnancy and lactation, despite no data supporting these restrictions being reported^98^. Another possible problem concerning breastfeeding could be an unsatisfactory milk supply caused by either combined hormonal contraceptives or reduction mammoplasty^97^.

However, the general consensus among researchers states that all the hurdles previously mentioned could be overcome, granted specific measures are taken, in order to assure at least 6 months of exclusive maternal milk diet for the infant, given the fact that the advantages of breastfeeding largely outweigh the possible risks.

## 5. Approach towards breastfeeding during COVID-19 pandemic

Given the scarcity of research regarding SARS-CoV-2 vertical transmission via breast milk, since the beginning of the pandemic there have been issued several guidelines concerning recommended approaches towards breastfeeding and mother-infant interaction in the cases of women tested positive for SARS-CoV-2. There have been two currents of opinion on this topic.

On one hand, right at the beginning of the outbreak in China, when little was known about the virus, most experts suggested that infants should be isolated from their mothers for at least 2 weeks, or until two consecutive RT-PCR tests yielded negative results^99^. Moreover, breastfeeding was discouraged, since there was no sufficient evidence that the virus can’t be spread via the milk. However, it was recommended that women expressed their milk which would later be given to the infants by third parties.

On the other hand, some major international organizations, such as the World Health Organization^100^, or UNICEF^101^, have recommended commencing breastfeeding right after parturition, following some clear protection measures. Therefore, asymptomatic or paucisymptomatic women were encouraged to breastfeed while constantly wearing a surgical mask throughout the entire process, properly washing their hands before and after handling the baby, sanitizing the breast area, as well as keeping a 2 meter distance from the infant when rooming in. In the cases of women who were experiencing more severe symptoms, isolation from the neonate was recommended, at least until an improvement of their condition, while being allowed nonetheless to express their milk in sterile containers and the infant being bottle fed with that milk. However, pasteurizing the milk has not been recommended, since it is known to inactivate an important part of the immune components of the milk^101,102^, such as antibodies that could play an important part in protecting the infant.

Since then, more research studies on the potential vertical transmission via breast milk were published, and it was understood that this means of spreading of the virus is rather unlikely. More and more health organizations are recommending and encouraging breastfeeding and skin-to-skin contact^103,104^.

## 6. Conclusion

In conclusion, it is likely to assume that vertical transmission via breast milk is a rather improbable route of transmission and spreading the virus. So far, the vast majority of milk samples were tested negative, while the few specimens that were tested positive for SARS-CoV-2 RNA may not have contained viable viral particles, according to some reports, seem to further tip the balance towards invalidating the hypothesis of viral spreading via maternal milk. Moreover, when taking into consideration the whole spectrum of benefits brought by breastfeeding, both for the neonate’s and mother’s evolution, the advantage far outweighs the potential risks. However, in order to obtain a definitive picture regarding the risks of SARS-CoV-2 transmission via breast milk, documented reports on the presence of replicative virus in cell culture from breast milk and infectivity in animal models are needed.
